# Study on the Freeze-Thaw Resistance of Concrete Pavements in Seasonally Frozen Regions

**DOI:** 10.3390/ma17081902

**Published:** 2024-04-19

**Authors:** Ruize Zhao, Chenglin Shi, Ruixin Zhang, Wensheng Wang, Huirong Zhu, Jing Luo

**Affiliations:** 1 College of Transportation Science and Engineering, Jilin Jianzhu University, Changchun 130118, China; zhaorzfly@163.com; 2 Kay Laboratory of Architectral Cold Climate Energy Management, Ministry of Education, Jilin Jianzhu University, Changchun 130118, China; 3 Highway Administration of Jilin Province, Changchun 130021, China; 4 College of Transportation, Jilin University, Changchun 130022, China; 5 College of Materials Science and Engineering, Jilin Jianzhu University, Changchun 130118, China

**Keywords:** seasonally frozen regions, concrete pavement, frost resistance, evaluation methods, salt freeze-thaw resistance

## Abstract

In seasonally frozen regions, concrete pavement is exposed to cycles of freeze-thaw and erosion from de-icing salt, which can lead to unfavorable service conditions and vulnerability to damage. This paper examines the compressive strength, flexural-tensile strength, abrasion resistance, permeability, and spacing factor of concrete, taking into account the impact of various curing conditions, de-icing salt solutions, and mass fractions on the concrete’s freeze-thaw resistance. Two test methods, the single-face method and the fast-freezing method, were used to comparatively analyze the freeze-thaw resistance of concrete. The analysis was based on the surface scaling, water absorption rate, mass loss rate, relative dynamic elastic modulus, and relative durability index. The results indicate that the presence of salt solution significantly worsened the degree of concrete damage caused by freeze-thaw cycles. The use of freeze-thaw media, specifically sodium chloride (NaCl), calcium chloride (${CaCl}_{2}$), and potassium acetate (KAc) at mass fractions of 5%, 4.74%, and 5%, respectively, had the greatest impact on the surface scaling of concrete. However, their effect on the water absorption rate was inconsistent. When the freeze-thaw medium was water, the concrete’s relative dynamic elastic modulus and relative durability index were 9.6% and 75.3% higher, respectively, for concrete cured in 20 °C—95% RH conditions compared to those cured in 0 °C—50% RH conditions. We propose a comprehensive relative durability index (${DF}_{w}$) by combining the results of two methods of freeze-thaw tests. The ${DF}_{w}$ of concrete cured in 0 °C—50% RH conditions was 83.8% lower than that of concrete cured in 20 °C—95% RH conditions when exposed to a freeze-thaw medium of 5% mass fraction NaCl solution. To evaluate the salt freeze-thaw resistance of concrete pavement, it is recommended to use surface scaling and ${DF}_{w}$ together.

## 1. Introduction

In China, over half of the country’s land area (53.5%) is covered by seasonal frozen regions [1,2]. These regions have harsh climates, characterized by long and cold winters. Due to the short construction period for concrete projects and the delivery period for highway projects, concrete pavement is often opened to traffic without proper curing. It is widely acknowledged that the curing conditions of concrete have a significant impact on cement hydration [3,4,5]. This, in turn, affects both the mechanical properties [6] and durability [7,8] of the material. Concrete pavement, which is exposed to the atmosphere and subjected to load and temperature stresses, is particularly vulnerable to deterioration caused by freeze-thaw cycles [9,10,11,12,13,14,15]. The damage to the durability of concrete that is not properly cured can be worsened by the combined effect of wheel loading and freeze-thaw cycles [16]. Therefore, it is essential to conduct a thorough evaluation of the frost resistance of concrete pavement under various curing conditions.

In structural design, the strength of concrete is crucial, but in many practical situations, durability takes precedence [17,18,19]. In seasonal frozen regions, the frost resistance of concrete pavement has a significant impact on its service life [20,21]. The freeze-thaw resistance of concrete may be affected if supplementary cementitious materials partially replace some of the cement [22]. Besheli et al. [23] found that replacing 15% of cement with natural zeolite in concrete pavement can improve its frost resistance. Zong et al. [24] discovered that the addition of ultra-fine supplementary cementitious materials can optimize the pore structure of concrete, leading to improved mechanical properties and durability. Similarly, Gonzalez et al. [25] and Yasien et al. [26] found that the use of nano-silica can increase the paste density of concrete, thereby enhancing its frost resistance. Incorporating appropriate amounts of fly ash and fibers can ensure favorable frost resistance of concrete in seasonally frozen regions [27,28]. However, it is important to note that Qiu et al. [29] found that the inclusion of fly ash led to the deterioration of the pore structure, resulting in reduced frost resistance, but it did promote the formation of tobermorite and C-A-S-H. Concrete pavements that incorporate supplementary cementitious materials are commonly used in practical engineering. Ensuring their durability remains a challenge.

De-icing salts are commonly used to melt ice and snow on concrete pavement in order to prevent traffic accidents caused by slippery winter snowfall. However, there is a concern that concrete pavement in seasonally frozen regions will be subjected to the combined effects of freeze-thaw cycles and de-icing salt erosion within 1–2 months after pouring. The structural performance of concrete pavement is approximately ten times more damaged by the combined effect of freeze-thaw cycles and de-icing salt than by freeze-thaw cycles alone [30]. Several theories have emerged since the last century that provide the basis for studying the salt freeze-thaw resistance of concrete. These include the Hydrostatic pressure theory [31], the Osmotic pressure theory [32], the Critical water saturation theory [33], the Cohesive scaling theory [34], and the Saturation and ice-formation pressure theory [35]. Recently, Qiao et al. [36] found that de-icing salts can cause significant leaching of calcium hydroxide, increasing the apparent porosity of the hydrate by approximately 23%. Duan et al. [37] found that concrete cracks extend inward and horizontally when the tensile stress at the ice-concrete composite interface exceeds the ultimate tensile strength of the concrete. Zhang et al. [38] and Yang et al. [39] discovered that the combined effect of de-icing salt erosion and freeze-thaw cycles can cause the internal microstructure in the transition zone of the concrete interface to loosen and degrade. In addition, numerous studies have shown that the application of a hydrophobic treatment to concrete surfaces can significantly improve its salt freeze-thaw resistance [40,41,42]. However, the hydrophobic layer formed on the concrete surface is susceptible to damage during freeze-thaw cycles [43,44].

To investigate the freeze-thaw resistance of concrete pavements in seasonally frozen regions, we examined several factors that influence it, such as curing conditions, de-icing salt solutions, and their mass fractions. The freeze-thaw properties were analyzed by comparing surface scaling, water absorption rate, mass loss rate, relative dynamic elastic modulus, and relative durability index using the single-sided freeze-thaw method and the rapid freeze-thaw method. Furthermore, this paper proposes an evaluation method for the salt freeze-thaw resistance of concrete by analyzing the evaluation indexes of current specifications and combining the results of test data. The aim is to provide a foundation for designing, constructing, and standardizing concrete pavement in seasonally frozen regions.

## 2. Materials and Methods

### 2.1. Materials and Mix Designs

Portland cement with a strength grade of 42.5 and fly ash classified as F class I were used as cementitious materials. The chemical compositions and physical properties of these materials are outlined in Table 1. The coarse aggregate consisted of 5–20 mm basalt crushed stone, and the fine aggregate was natural river sand. Table 2 displays the main properties of the coarse and fine aggregates. Additionally, to enhance frost resistance, an air-entraining agent was added, and the air content of the concrete was controlled at 4.6 ± 0.2. A polycarboxylate superplasticizer was used to improve the workability of the concrete, resulting in a consistent slump of 75 ± 10 mm in each batch of fresh concrete. Please refer to Table 3 for specific details on the mix proportions of the concrete.

### 2.2. Specimen Preparation and Curing 

The concrete mixing process utilized the CABR-HJS60 concrete double horizontal shaft mixer. After pouring the concrete mix into the molds, the surface was smoothed following 45 s ± 15 s of vibration. The molded concrete was then kept indoors for 24 h ± 2 h at a temperature of 15 °C ± 3 °C before being removed from the molds and placed into various environmental curing boxes. Three distinct curing conditions were implemented: Standard curing (SC), Intermediate curing (IC), and Low-temperature curing (LC). The curing conditions for each specimen are outlined in Table 4.

### 2.3. Test Methods

#### 2.3.1. Important Properties of Concrete Pavement Test

##### Compressive Strength and Flexural-Tensile Strength Tests

The compressive strength and flexural-tensile strength of concrete cured for 28 days under different conditions were tested, with reference to JTG3420-2020 [45]. Cubic specimens measuring 100 mm × 100 mm × 100 mm and prismatic specimens measuring 100 mm × 100 mm × 400 mm were used to measure the compressive and flexural-tensile strength, respectively. Each group consisted of three specimens. The loading rates for the compressive strength test and the flexural-tensile strength test were set at 5 KN/s and 0.5 KN/s, respectively, until the specimens were destroyed.

##### Abrasion Resistance Test

The abrasion resistance test was conducted on concrete cube specimens that measured 150 mm × 150 mm × 150 mm. Each group comprised three specimens. After 27 days of different curing conditions, the specimens were removed from the environmental curing box. The surface water was dried, and they were left to dry naturally in indoor air for 12 h. Subsequently, they were placed in an oven at 60 °C and baked until they reached a constant weight. After removing the specimens from the oven, they were allowed to cool to room temperature. Brush the surface to remove any dust. Brush the surface clean of any dust and weigh the specimen. The specimen was ground under a 200 N load for 30 revolutions. Afterward, it was removed and brushed to eliminate surface dust before being weighed. Next, grind the specimen for an additional 60 revolutions under a 200 N load. Finally, calculate the wear amount per unit area with reference to JTG3420-2020 [45].

##### Rapid Chloride Permeability Test

The charge passed method was used to conduct the rapid chloride permeability test in accordance with ASTM C 1202 [46]. Each group consisted of three cylindrical concrete specimens with dimensions of φ100 mm × 50 mm. The specimens were initially cured under different conditions for 28 days and then left to air dry. Silica gel was applied to the cylindrical sides of the specimens, ensuring that any coating holes were filled. The specimens were vacuum-saturated and immersed in a vacuum saturation machine. Next, we checked the seal between the specimen and the test tank. The test setup included adding a 3.0% mass concentration NaCl solution and a 0.3 mol/L molar concentration sodium hydroxide (NaOH) solution to both sides of the test tank. The negative electrode was connected to the NaCl solution, while the positive electrode was connected to the NaOH solution. A 60 V DC voltage was applied and readings were taken every 15 min using an automated data collection device. After 6 h, the sample was removed from the test cell and the charge passed by the sample was measured and recorded.

##### Spacing Factor Test

The specimens were processed as follows according to ASTM C 457 [47]: First, each original specimen with dimensions of 100 mm × 100 mm × 100 mm was cut into prismatic test specimens measuring 100 mm × 100 mm × 20 mm along the direction of the vertical molding surface. Next, the specimens were washed with anhydrous ethanol. Finally, the forming surface of each specimen was ground using a fixed diamond mill with two different grain sizes: 250 μm and 74 μm. After the grinding process, the specimen underwent sequential polishing with 400, 800, and 1200 mesh polishing paste, each containing 10% diamond content. Once the grinding and polishing steps were completed, the specimen was dried and coated with fluorescent dye. Subsequently, we tested the specimens under different maintenance conditions using a fully automated air-void analyzer. The color photographs were converted to grayscale images by the analyzer. The background inhomogeneity was corrected through a filtering process to obtain the final binarized images for analysis and calculation.

#### 2.3.2. Freeze-Thaw Test

##### Single-Sided Freeze-Thaw Test

The specimens measuring φ200 mm × 80 mm underwent a single-sided freeze-thaw test. Each group consisted of three specimens. The specimens were taken out of environmental curing boxes and all surfaces, except for the test surface and the top surface parallel to it, were sealed using epoxy resin. The specimens were submerged in the solution to a depth of 5 mm for a pre-absorption time of 4 days. At 28 days of age, the molding surface was used as the test surface for the single-sided freeze-thaw test. The test employed freeze-thaw media with varying mass fractions of NaCl solution (1%, 3%, 5%, and 7%), ${CaCl}_{2}$ solution (0.95%, 2.85%, 4.74%, and 6.64% with the same molar concentration of Cl ions as the NaCl solution), and Kac solution (1%, 3%, 5%, and 7%). A control group was also established using water as the freeze-thaw media, as presented in Table 5.

The freeze-thaw cycle test temperature started at 20 °C and gradually decreased to −20 °C at a rate of 10 °C/h. It was then held at −20 °C for 3 h before being gradually increased to 10 °C at a rate of 10 °C/h and held for 1 h. The specimen was subjected to an ultrasonic bath for 3 min after each 5 freeze-thaw cycles with the test surface facing down. The specimen was then weighed and the mass recorded after wiping off any water. The surface scaling was then dried in an oven at 105 °C for 24 h, and the total mass of the surface scaling was weighed after cooling. The surface scaling and water absorption rate of the specimen were measured every five freeze-thaw cycles. Equations (1) and (2) were used to calculate the surface scaling and water absorption rate, respectively. The test was terminated when one of the following two conditions was met:

1. After 30 freeze-thaw cycles.
2. Surface scaling exceeding 1000 ${g/m}^{2}$.

(1) $$m_{n}=\frac{\sum \mu_{s}}{A}\times 10^{6}\;$$

where $m_{n}$ is the surface scaling per unit area after *n* freeze-thaw cycles (${g/m}^{2}$); $\mu_{s}$ is the mass of specimen flaking obtained at each test gap, accurate to 0.01 g (g); A is the surface area of a single tested specimen ($m^{2}$).

(2) $${∆w}_{n}=\frac{w_{n}-w_{1}+\sum \mu_{s}}{w_{0}}\times 100\%\;$$

where ${∆w}_{n}$ is the water absorption rate of the specimen after *n* freeze-thaw cycles (%); $w_{0}$ is the net mass of the specimen in the dry state before sealing (excluding the mass of the side seals), accurate to 0.1 g (g); $w_{n}$ is the mass of the specimen after *n* freeze-thaw cycles (including the mass of the side seals), accurate to 0.1 g (g); $w_{1}$ is the mass of the specimen (including the mass of the side seals) before it is saturated with water after sealing, accurate to 0.1 g (g).

##### Rapid Freeze-Thaw Test

For the rapid freeze-thaw test, three concrete specimens measuring 100 mm × 100 mm × 400 mm were used in each group. Prior to the test, the cured specimens were immersed for four days. After immersion, the surface water was wiped off with a damp cloth, and the transverse fundamental frequency was measured. The mass was also weighed and used as the initial value for evaluating the concrete’s freeze-thaw resistance. During the freeze-thaw test, the specimens were immersed in an elastic rubber test tank filled with a solution of water and various salts. The freeze-thaw media was composed of water, a 5% mass fraction NaCl solution, a 4.74% mass fraction ${CaCl}_{2}$ solution, and a 5% mass fraction KAc solution. The specimen underwent freeze-thaw cycles with controlled temperatures of −18 °C and 5 °C at the center, respectively. Each cycle lasted for 3 h. The transverse fundamental frequency of the specimen was measured and recorded once every 25 freeze-thaw cycles. After each test, the specimen was reloaded into the specimen box filled with water or various salts, and the test continued. The mass loss rate, relative dynamic elastic modulus, and relative durability index of the concrete were calculated using Equations (3)–(5), respectively. The test was terminated when one of the following two conditions was met:

1. Mass loss rate exceeding 5%.
2. Relative dynamic elastic modulus decreased to less than 60%.

(3) $$P=\frac{f_{n}^{2}}{f_{0}^{2}}\times 100\%\;$$

In which, *P* is the relative dynamic elastic modulus after *n* freeze-thaw cycles (%), *D* is the transverse fundamental frequency after *n* freeze-thaw cycles (Hz), and *S* is the transverse fundamental frequency before the test (Hz).

(4) $$W_{n}=\frac{m_{0}-m_{n}}{m_{0}}\times 100\%\;$$

In which, $W_{n}$ is the mass loss rate after *n* freeze-thaw cycles (%); $m_{0}$ is the specimen mass before the freeze-thaw test (kg); and $m_{n}$ is the mass after *n* freeze-thaw cycles (kg).

(5) $$K_{n}=\frac{P\times N}{300}\;$$

In which, $K_{n}$ is the relative durability index after *n* freeze-thaw cycles (%); N is the number of freeze-thaw cycles at the end of the test; and P is the relative dynamic elastic modulus after *n* freeze-thaw cycles (%).

## 3. Results and Discussion

### 3.1. Material Properties

**Figure 1:**
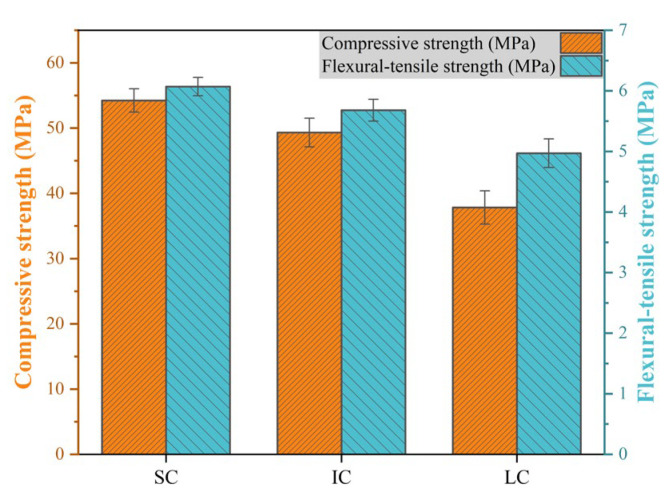
Compressive strength and flexural-tensile strength of concrete under various curing conditions.

Figure 1 displays the compressive and flexural-tensile strengths of SC, IC, and LC concrete. LC concrete exhibited a significantly lower compressive strength, being 30.2% and 23.3% lower than SC and IC concrete, respectively. Furthermore, SC and IC concrete demonstrated 22.1% and 14.3% higher flexural-tensile strengths than LC concrete, respectively. It is important to note that inadequate curing conditions may lead to a decrease in the compressive and flexural-tensile strengths of concrete.

**Figure 2:**
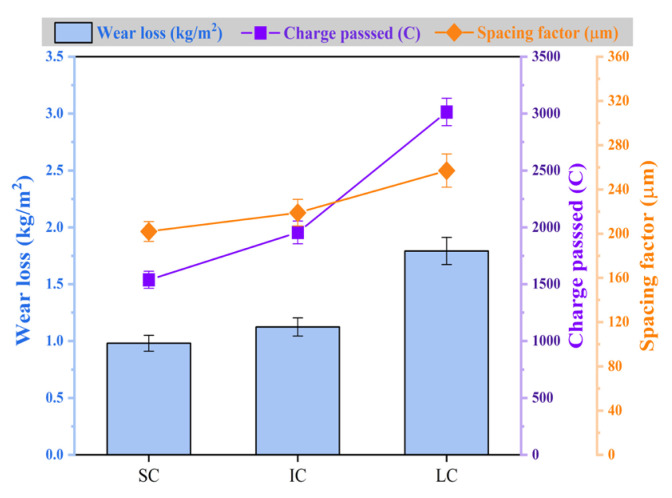
Abrasion resistance, permeability, and spacing factor of concrete under various curing conditions.

Improving the abrasion resistance of concrete is crucial for extending the pavement’s service life and ensuring safe travel while meeting mechanical property requirements. According to Figure 2, LC concrete had significantly higher unit area wear than SC and IC concrete, with 82.9% and 59.4% higher wear, respectively. To achieve excellent abrasion resistance in concrete pavement, it is necessary to ensure proper curing temperature and humidity. Factors affecting concrete durability are primarily related to fluid transport through the material [18,48,49]. LC concrete exhibited a 95.9% higher electrical flux than SC concrete and a 54.1% higher electrical flux than IC concrete. Additionally, the spacing factor of LC concrete was 27.2% higher than that of SC concrete and 17.4% higher than that of IC concrete. This variation may be attributed to differences in cement hydration and the distribution of hydration products in the concrete under varying curing conditions, which impact the pore structure and connectivity of the concrete.

### 3.2. Freeze-Thaw Resistance

#### 3.2.1. Single-Sided Freeze-Thaw Method

##### Surface Scaling

**Figure 3:**
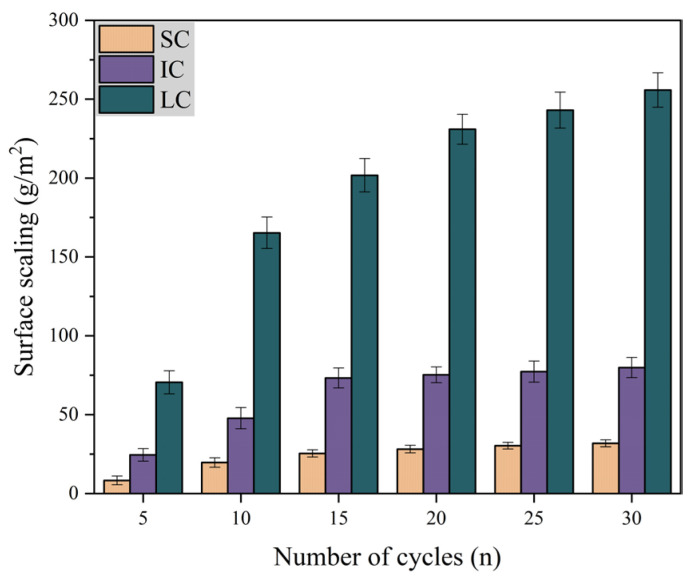
Surface scaling with the number of freeze-thaw cycles in water.

Figure 3 shows the variation of surface scaling with the number of freeze-thaw cycles under different curing conditions when the freeze-thaw medium is water. It is evident that SC concrete had the lowest surface scaling and LC concrete had the highest surface scaling. The surface scaling of SC concrete and IC concrete remained almost unchanged after 15 freeze-thaw cycles. From 20–30 freeze-thaw cycles, the surface scaling of LC concrete increased by only 10.8%. The data indicates that the concrete surface scaling is lower when exposed to single-sided freeze-thaw cycles with water as the medium.

**Figure 4:**
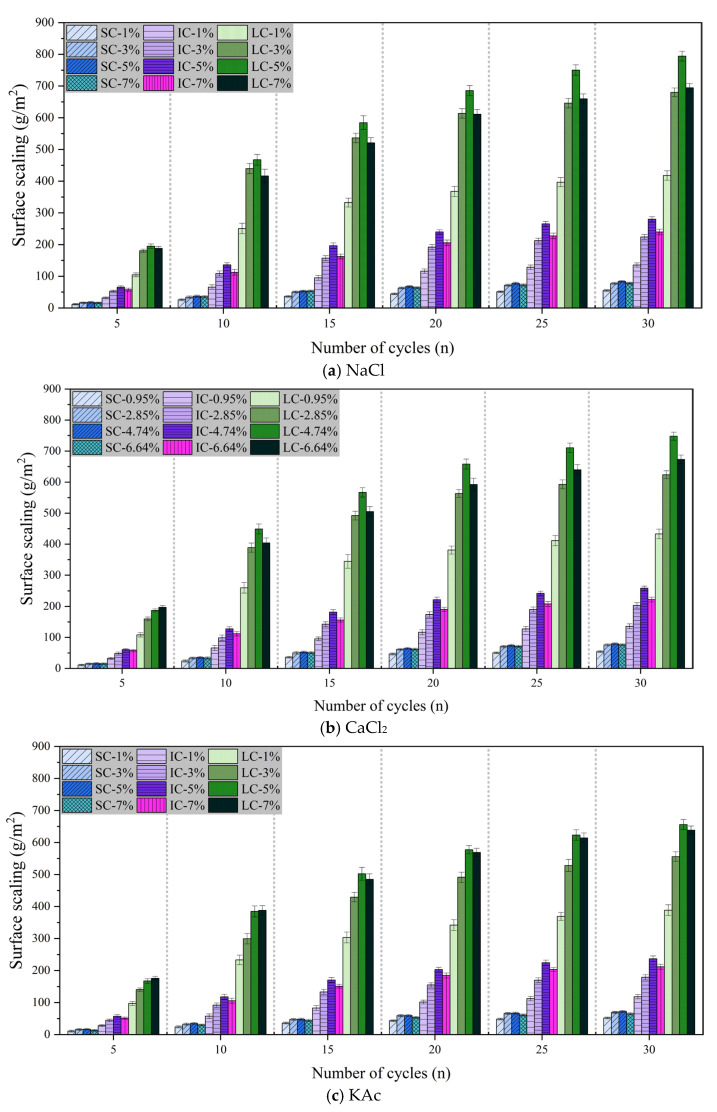
Surface scaling with the number of freeze-thaw cycles in salt solutions.

The durability of concrete pavement can be reduced due to spalling of hardened mortar and aggregates caused by the combined effect of salt solution and freeze-thaw cycles [11,50,51,52]. Figure 4a–c demonstrate the surface scaling of concrete with the number of freeze-thaw cycles under different curing conditions when the freeze-thaw medium is NaCl, CaCl, and KAc solution, respectively.

Figure 4a displays the surface scaling of concrete with freeze-thaw cycles under various curing conditions when the freeze-thaw cycle medium is NaCl solution. The surface scaling of the SC concrete was the lowest and had the least variation with the number of freeze-thaw cycles. The lowest surface scaling was observed after 30 freeze-thaw cycles with a NaCl solution mass fraction of 1%. However, there was no significant difference in the surface scaling of concrete for NaCl solution mass fractions of 3%, 5%, and 7%. The surface scaling of SC concrete and IC concrete was highest when the mass fraction of NaCl solution was 5%. After 30 freeze-thaw cycles, the surface scaling of the LC concrete increased by 90.1%, 16.8%, and 14.4% when the mass fraction of NaCl solution was 5%, compared to 1%, 3%, and 7%, respectively.

Figure 4b illustrates that the surface scaling of concrete is comparable when exposed to freeze-thaw cycles in ${CaCl}_{2}$ solution and NaCl solution. Figure 4c displays the variation of surface scaling of concrete with freeze-thaw cycles under different curing conditions when the freeze-thaw cycle medium is KAc solution. After 30 freeze-thaw cycles, SC concrete exhibited the lowest surface scaling and the smallest difference between freeze-thaw media. For both IC concrete and LC concrete, the surface scaling was significantly lower when the mass fraction of KAc solution was 1% compared to when it was 3%, 5%, and 7%. It is worth noting that after 30 freeze-thaw cycles, the surface scaling of LC concrete was highest when the KAc solution mass fraction was 5%, but only 2.7% higher than when it was 7%.

**Figure 5:**
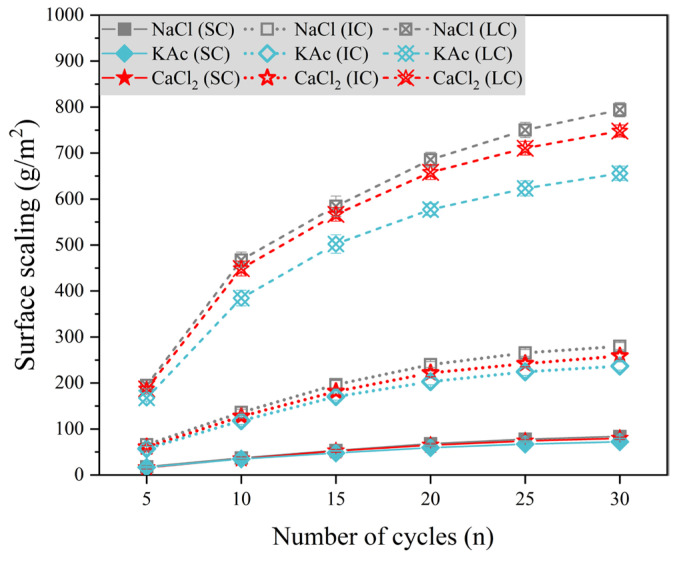
Surface scaling with the number of freeze-thaw cycles at salt solutions of 5% mass fraction of NaCl solution, 4.74% mass fraction of CaCl_2_ solution, and 5% mass fraction of KAc solution.

To compare and analyze the effects of various curing conditions and salt solutions on concrete surface scaling, we utilized 5% NaCl solution, 4.74% ${CaCl}_{2}$ solution, and 5% KAc solution as examples for further analysis. Figure 5 illustrates the variation of concrete surface scaling with the number of freeze-thaw cycles under three different salt solution conditions. The surface scaling of SC concrete was significantly lower, while there was no significant difference in the concrete surface scaling of the three freeze-thaw media. For IC concrete, there was no significant difference in the surface scaling of concrete during the first 10 freeze-thaw cycles with NaCl, ${CaCl}_{2}$, and KAc solutions as freeze-thaw media. After 15 freeze-thaw cycles, the surface scaling of concrete was slightly higher when the freeze-thaw medium was NaCl solution, while it was slightly lower when the freeze-thaw medium was KAc solution. After 30 freeze-thaw cycles, the surface scaling of LC concrete was 21.1% and 14.0% higher when the freeze-thaw medium was NaCl solution and ${CaCl}_{2}$.

Comparison of Figure 3 shows that the degree of concrete damage resulting from salt solution and freeze-thaw cycles was significantly greater than that caused by a single freeze-thaw cycle. This effect was particularly evident for LC concrete. Among the solutions tested, NaCl, ${CaCl}_{2}$, and KAc had the most severe impact on concrete surface scaling at mass fractions of 5%, 4.74%, and 5%, respectively. According to the Saturation and ice-formation pressure theory [35], the presence of a salt solution can significantly increase the internal saturation of concrete, resulting in increased surface scaling. However, it can also decrease the rate of solution icing expansion and icing pressure, increase the critical saturation of the solution to produce icing pressure, and mitigate the surface scaling. The combination of these two factors results in a low mass fraction of salt solution, which in turn produces the highest icing pressure and leads to the most severe damage to the concrete.

##### Water Absorption Rate

**Figure 6:**
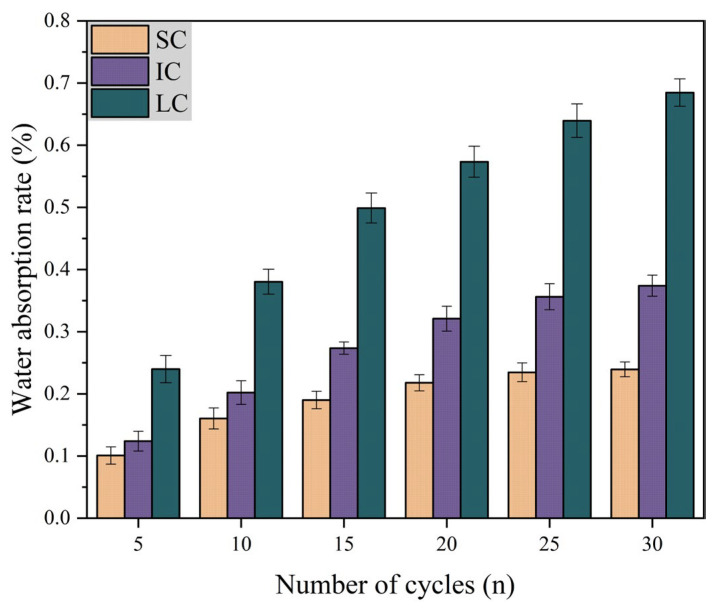
Water absorption rate with the number of freeze-thaw cycles in water.

Figure 6 shows the variation of water absorption rate with the number of freeze-thaw cycles under different curing conditions when the freeze-thaw medium is water. It is found that LC concrete exhibited significantly higher water absorption rate than SC concrete and IC concrete. After 15–30 freeze-thaw cycles, the water absorption rate of LC concrete was found to be 162.7% to 186.1% higher than that of SC concrete, while the water absorption rate of LC concrete was 82.4% to 83.0% higher than that of IC concrete. These results suggest that as the number of freeze-thaw cycles increases, the water absorption rate of SC concrete increases to a lesser extent than that of LC concrete, while the water absorption of IC concrete increases to a similar extent as that of LC concrete.

**Figure 7:**
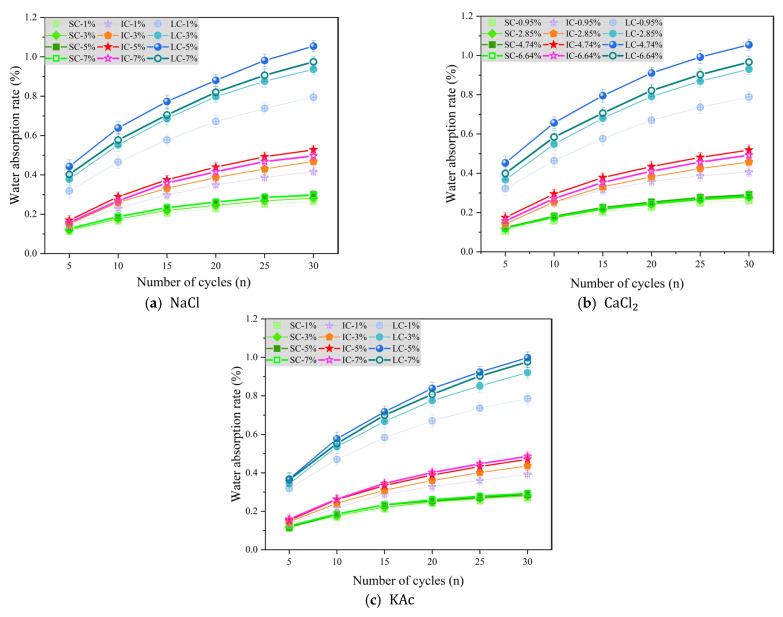
Water absorption rate with the number of freeze-thaw cycles in salt solutions.

Figure 7a–c display the change in concrete water absorption rate as a function of the number of freeze-thaw cycles under various curing conditions when the freeze-thaw medium is NaCl, ${CaCl}_{2}$, and KAc solutions, respectively.

Figure 7a shows the variation in water absorption rate of concrete with the number of freeze-thaw cycles under different curing conditions when the freeze-thaw cycle medium is NaCl solution. The water absorption rates of SC concrete with NaCl solution mass fractions of 5% and 7% were slightly higher than those of concrete with NaCl solution mass fractions of 1% and 3%, but the difference was not significant. The water absorption rate of IC concrete was highest when the mass fraction of NaCl solution was 5%. After 30 freeze-thaw cycles, the water absorption rate was 26.7%, 12.8%, and 6.2% higher when the NaCl solution mass fraction was 5% compared to 1%, 3%, and 7%, respectively. Furthermore, LC concrete had significantly higher water absorption rate than SC and IC concrete. After 30 freeze-thaw cycles, the water absorption rate of concrete with a NaCl solution mass fraction of 5% was higher than that of concrete with NaCl solution mass fractions of 1%, 3%, and 7% by 32.6%, 12.5%, and 8.1%, respectively. When the freeze-thaw medium is a ${CaCl}_{2}$ solution, the water absorption rate of concrete under each curing condition has a similar trend compared to when the freeze-thaw medium is a NaCl solution, as shown in Figure 7b. This suggests that chloride ions in de-icing salt may be the main cause of freeze-thaw damage in concrete.

Figure 7c displays the change in concrete water absorption rate with the number of freeze-thaw cycles when using KAc solution as the freeze-thaw cycle medium. It is clear that the water absorption rate of SC concrete was less affected by the solution mass fraction. On the other hand, the water absorption rate of the IC concrete was the highest when the mass fraction of the KAc solution was 7%. After undergoing 30 freeze-thaw cycles, the water absorption rate of concrete with a 7% KAc solution mass fraction was higher than that of concrete with 1%, 3%, and 5% KAc solution mass fractions by 23.8%, 11.4%, and 3.3%, respectively. Furthermore, in regards to LC concrete, the water absorption rate only increased by 2.2% when a 5% mass fraction of KAc solution was used compared to a 7% mass fraction. It was also found to be 27.1% and 8.4% higher than LC concrete when a 1% and 3% mass fraction of KAc solution was used, respectively.

**Figure 8:**
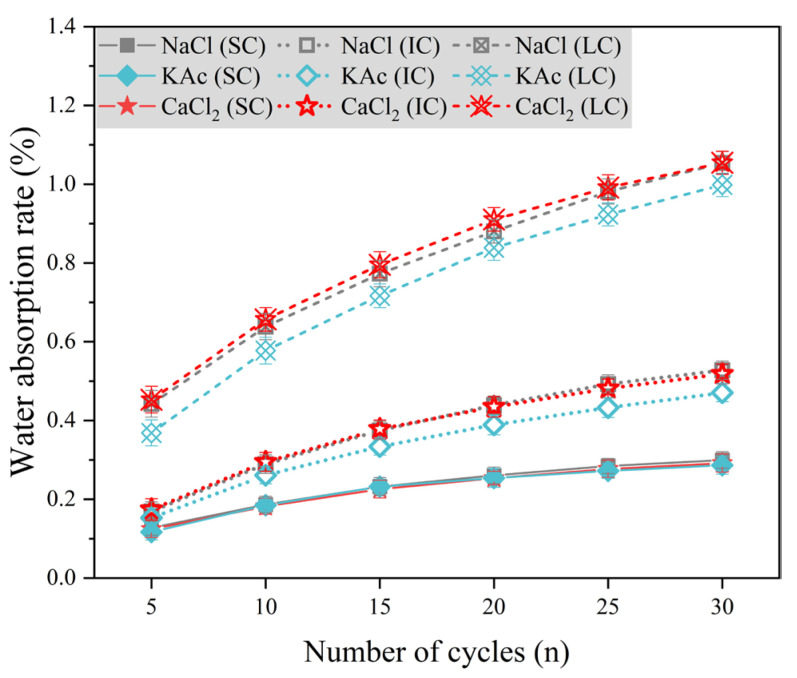
Water absorption rate with the number of freeze-thaw cycles at salt solutions of 5% mass fraction of NaCl solution, 4.74% mass fraction of ${CaCl}_{2}$ solution and 5% mass fraction of KAc solution.

Figure 8 displays the water absorption rate variation of concrete with the number of freeze-thaw cycles, using 5% mass fraction of NaCl solution, 4.74% mass fraction of ${CaCl}_{2}$ solution, and 5% mass fraction of KAc solution as the freeze-thaw medium. The water absorption rate of SC concrete was significantly lower than that of IC and LC concrete. Additionally, there was little difference in the water absorption rate of SC concrete under the three salt solution conditions. The water absorption rates of IC concrete and LC concrete were similar when exposed to NaCl and ${CaCl}_{2}$ solutions as freeze-thaw mediums. However, these rates were higher compared to concrete exposed to KAc solution. For instance, after 30 freeze-thaw cycles, the water absorption rates of IC concrete and LC concrete were 12.1% and 5.6% higher than those of concrete exposed to KAc solution, respectively. The results indicate that the water absorption rate of IC and LC concrete is reduced under KAc solution conditions compared to NaCl and ${CaCl}_{2}$ solutions as freeze-thaw mediums. However, the change in water absorption rate for SC concrete is not significant.

In summary, after being subjected to a single-sided freeze-thaw cycle and salt solution, it was observed that the surface scaling and water absorption rate of SC concrete were lower and less affected by the freeze-thaw medium. In contrast, the surface scaling and water absorption rate of LC concrete were significantly higher. This difference may be attributed to the pore structure and connectivity of the concrete itself. In addition, the presence of salt on concrete surfaces can cause water to migrate to the surface, which, if frozen, can create destructive pressure. It is important to note that the development of pores continues in the direction of stress concentration towards neighboring pores [36,53]. This can damage the internal pore structure of the concrete and generate microcracks on the surface and at deeper depths. Additionally, certain grains may lose their bonding properties, resulting in an increase in capillary porosity and connectivity. As a result, the pore structure and connectivity of LC concrete are increased, leading to greater surface scaling and water absorption rate.

#### 3.2.2. Rapid Freeze-Thaw Method

**Figure 9:**
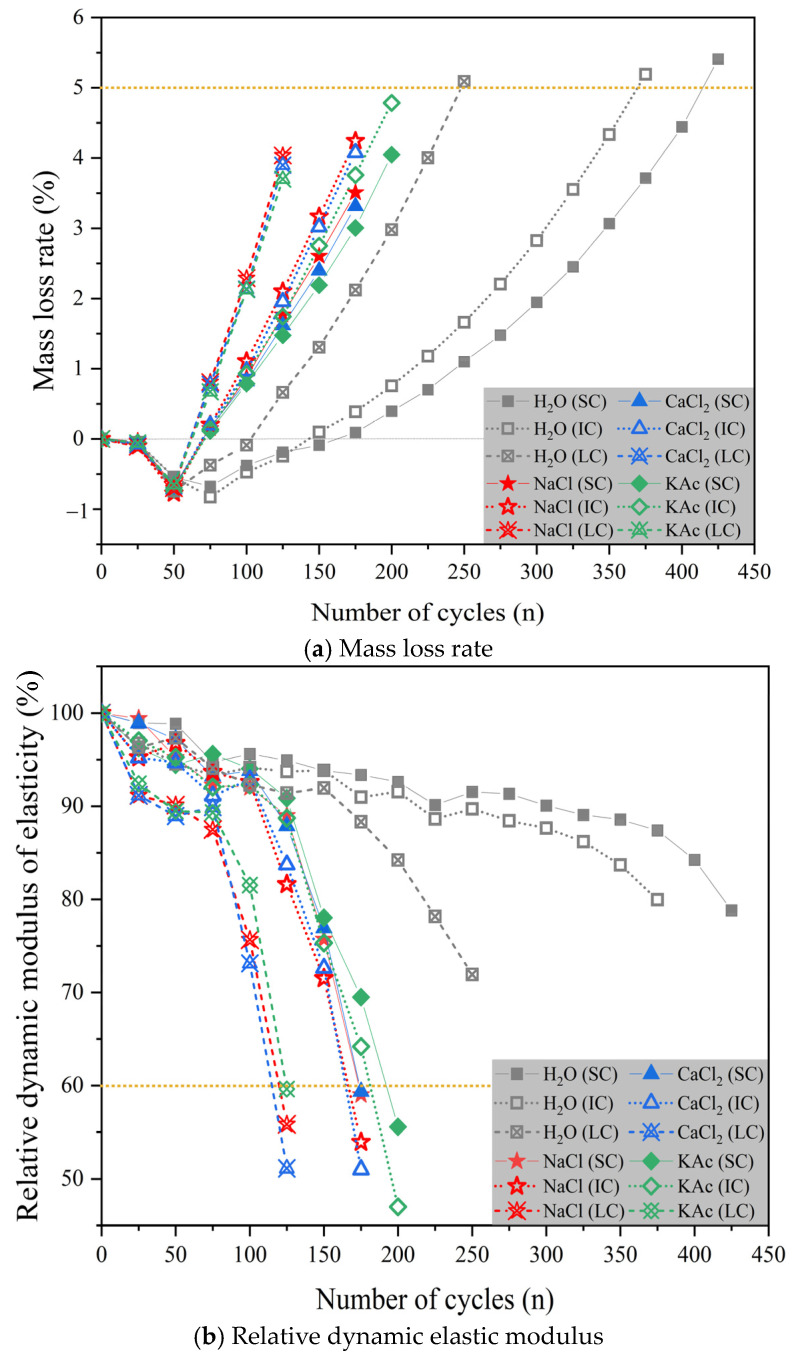

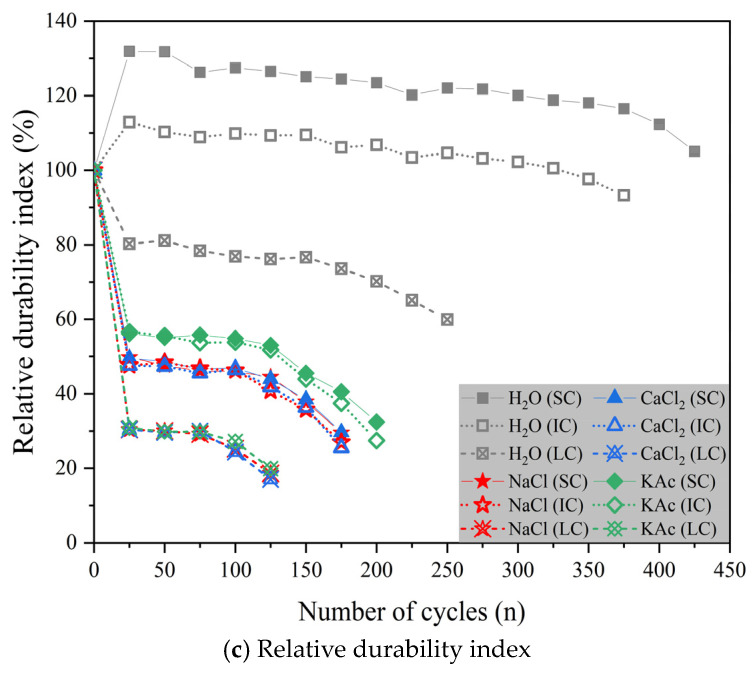
Mass loss rate, relative dynamic elastic modulus, and Relative durability index with the number of freeze-thaw cycles.

Figure 9a–c show the changes in mass loss rate, relative dynamic elastic modulus, and relative durability index of concrete with the number of freeze-thaw cycles under different curing conditions when the freeze-thaw medium is water and NaCl, ${CaCl}_{2}$, and KAc solutions, respectively.

Based on the data presented in Figure 9a, it is clear that the use of water as the freeze-thaw medium resulted in mass loss rates of over 5% for SC concrete and IC concrete after 425 and 375 cycles, respectively, while LC concrete reached 5% after only 250 cycles. When NaCl solution and ${CaCl}_{2}$ solution were used as the freeze-thaw medium, the mass loss rate of LC concrete increased significantly faster than that of SC concrete and IC concrete. When KAc solution was used as the freeze-thaw medium, the mass loss rate of SC concrete and IC concrete was lower compared to when NaCl solution and ${CaCl}_{2}$ solution were used. However, the difference in the mass loss rate of LC concrete was not significant. During the initial stage of the freeze-thaw cycle, all concrete mass loss rates decreased. However, as the cycle progressed, the concrete mass loss rate gradually increased. This phenomenon occurs because the concrete absorbs more water than it spalls during the preliminary stage of the freeze-thaw cycle. As the cycle progresses, microcracks develop in the outer layer of the concrete and continue towards the inner part of the specimen. This results in an increase in the rate of mass loss as the amount of spalling material increases.

Figure 9b illustrates that when the freeze-thaw medium is water, the relative dynamic elastic modulus of concrete at the end of the test follows the order: IC concrete > SC concrete > LC concrete. The relative dynamic elastic modulus of IC concrete and SC concrete was 11.2% and 9.6% higher than that of LC concrete, respectively. When the freeze-thaw medium was composed of NaCl and ${CaCl}_{2}$ solutions, the relative dynamic elastic modulus of both SC concrete and IC concrete decreased to less than 60% after 175 freeze-thaw cycles. However, when the freeze-thaw medium was KAc solution, the relative dynamic elastic modulus of both SC concrete and IC concrete decreased to less than 60% after 200 freeze-thaw cycles. Additionally, for LC concrete, the relative dynamic elastic modulus decreased to below 60% after only 125 freeze-thaw cycles when exposed to NaCl, ${CaCl}_{2}$, and KAc solutions as freeze-thaw media. It is important to note that the relative dynamic elastic modulus of concrete experiences a significant decrease during the later stages of the freeze-thaw cycle test when salt solution is used as the freeze-thaw medium. Therefore, the relative dynamic elastic modulus may not be entirely accurate in evaluating the coupling impact of salt solution and freeze-thaw cycles in the rapid freeze-thaw method.

Figure 9c displays the relative durability indices of each concrete group during freeze-thaw cycles using water as the medium. The SC concrete had a 75.3% higher relative durability index than the LC concrete at the end of the test. Table 6 presents the mass loss rate, relative dynamic elastic modulus, and relative durability index of concrete specimens for each test group at the end of the test. The durability index of concrete is significantly higher when exposed to freeze-thaw in water compared to salt solution. Frost resistance is closely related to the pore structure and connectivity of concrete [20,54,55,56,57]. Therefore, imperfect curing of concrete pavement may negatively affect the pore structure and connectivity, leading to reduced frost resistance. When water is used as the freeze-thaw medium, the relative dynamic elastic modulus of SC concrete, IC concrete, and LC concrete remains higher than 60%, even when the mass loss rate exceeds 5%. This is shown in combination with Figure 9a,b. However, if a salt solution is used as the freeze-thaw medium, the relative dynamic elastic modulus of each concrete group drops below 60% when the mass loss rate is not more than 5%. The study suggests that the freezing zone at the end of the test remains primarily in the water-saturated layer outside the concrete specimen when water is used as the freeze-thaw medium. In contrast, when a salt solution is used, the microcracks generated by the freeze-thaw cycle at the end of the test have extended into the interior of the specimen.

### 3.3. Comprehensive Relative Durability Index

Cement and concrete standards should provide clear and objective guidelines for most applications with low or moderate requirements. Additionally, they should offer appropriate and safe methods for assessing durability in severe conditions [58]. The water absorption rate in the single-sided freeze-thaw method can be used to evaluate the pore structure and capillary connectivity of concrete, as well as the effect of freeze-thaw cycles and salt solution coupling on it. However, concrete damage is the result of both surface and internal damage [38,39,52]. Therefore, a single value of water absorption rate does not fully reflect the degree of damage to the concrete interior caused by freeze-thaw cycles. Additionally, the current standard does not provide meaningful index requirements for water absorption rate.

Combined with the discussion of the test data of the rapid freeze-thaw method in Section 3.2.2, the relative dynamic elastic modulus in the rapid freeze-thaw method can better characterize the internal crack development of concrete when the freeze-thaw medium is water. Therefore, this paper proposes a comprehensive relative durability index (${DF}_{w}$) to evaluate the salt freeze-thaw resistance of concrete pavement. The ${DF}_{w}$ is based on the water absorption rate in the single-sided freeze-thaw method under different curing conditions, different salt solutions, and different mass fractions. It is also combined with the relative dynamic elastic modulus in the rapid freeze-thaw method under different curing conditions when the freeze-thaw medium is water. The comprehensive relative durability index (${DF}_{w}$) is calculated using the following Equation (6):

(6) $${DF}_{w}=[(P\times N)/N_{max}\;]/{∆w}_{n}\;$$

where ${DF}_{w}$ is the comprehensive relative durability index; P is the relative dynamic elastic modulus; N is the maximum number of freeze-thaw cycles of the specimen; $N_{max}$ is the maximum number of freeze-thaw cycles of all specimens in the test; and ${∆w}_{n}$ is the water absorption rate.

**Figure 10:**
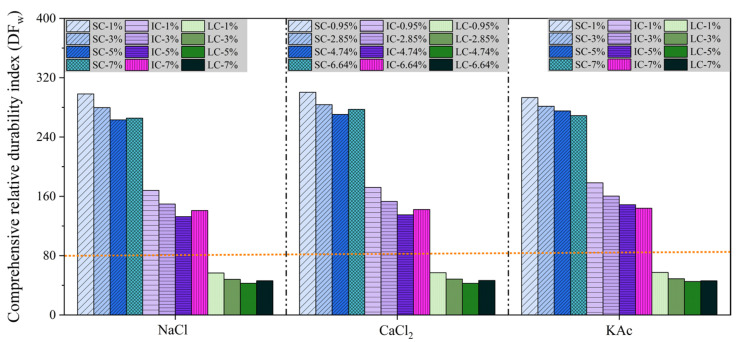
Comprehensive relative durability index (${DF}_{w}$) of concrete.

Figure 10 shows the ${DF}_{w}$ of concrete under various curing conditions, salt solutions, and mass fractions of freeze-thaw medium. The ${DF}_{w}$ of each concrete was significantly higher when the mass fraction of freeze-thaw medium was 1%. The lowest ${DF}_{w}$ for SC and IC concretes was observed with NaCl solution at 5%, ${CaCl}_{2}$ solution at 4.64%, and KAc solution at 7% mass fractions. For instance, when the freeze-thaw medium was a 5% mass fraction NaCl solution, the ${DF}_{w}$ of LC concrete was 83.8% lower than that of SC concrete and 67.8% lower than that of IC concrete. These results suggest that the curing conditions have a much greater impact on the ${DF}_{w}$ of concrete than the freeze-thaw medium and its mass fraction. Additionally, the salt freeze-thaw resistance of concrete can be evaluated by means of a mass fraction ranging from 3% to 7%. The recommended baseline value for assessing the superiority of salt freeze-thaw resistance of concrete pavement is 80. However, it should be noted that further research is needed to determine the accuracy of this index due to the limited amount of test data in this study.

To evaluate the salt freeze-thaw resistance of concrete pavement in seasonal frozen regions, it is recommended to conduct both the single-sided freeze-thaw method and the rapid freeze-thaw method. The paper suggests using surface scaling and DF for this purpose.

## 4. Conclusions

The study comprehensively explored and evaluated the freeze-thaw resistance of concrete in seasonal frozen regions based on its actual construction and service conditions. The following are the main conclusions:

1. The degree of concrete damage caused by freeze-thaw cycles was significantly worsened by the presence of salt solution. The damage to the concrete under 20 °C—95% RH curing conditions caused by the combined effect of freeze-thaw and salt solution was significantly lower than that of concrete under 0 °C—50% RH curing conditions. To ensure the freeze-thaw resistance of concrete pavement in seasonally frozen regions, it is necessary to develop a design and curing program that considers the climatic conditions.
2. The surface scaling of concrete was most affected by the freeze-thaw media NaCl, ${CaCl}_{2}$, and KAc, at mass fractions of 5%, 4.74%, and 5%, respectively. However, these media did not have a consistent effect on the water absorption rate. The use of KAc solution as a freeze-thaw medium resulted in reduced surface scaling and water absorption rate for concrete under 5 °C—70% RH curing condition and 0 °C—50% RH curing condition when compared to NaCl and ${CaCl}_{2}$ solutions.
3. Using the rapid freeze-thaw method with water as the medium, the freeze-thaw cycle test process was controlled by the mass loss rate. The freezing zone at the end of the test existed mainly in the water-saturated layer outside the specimen, and microcracks did not develop inside the specimen. In addition, when the freeze-thaw medium was a salt solution, the freeze-thaw cycle process was controlled by the relative dynamic modulus of elasticity, and the inside of the specimen was already damaged at the end of the test.
4. The rapid freeze-thaw method is more effective in evaluating the frost resistance of concrete when the freeze-thaw medium is water. However, it may not accurately compare the effects of the combined action of salt solution and freeze-thaw cycles on concrete.
5. The comprehensive relative durability index (${DF}_{w}$) was proposed based on the water absorption rate in the single-sided freeze-thaw method and the relative dynamic elastic modulus in the rapid freeze-thaw method. To assess the salt freeze-thaw resistance of concrete pavement, it is recommended to use both the surface scaling and the ${DF}_{w}$.

## Figures and Tables

**Table 1:** Chemical composition and physical properties of cement and fly ash.

Chemical Composition	Cement	Fly Ash	Physical Properties	Cement	Fly Ash
${SiO}_{2}$	22.76	62.219	Specific surface (m^2^/kg)	331	544
${Al}_{2}O_{3}$	5.45	20.203	Density (g/cm^3^)	3.1	2.40
${Fe}_{2}O_{3}$	3.15	2.915	Initial setting time (min)	145	-
CaO	60.42	8.906	Final setting time (min)	205	-
MgO	3.05	0.815	3 d Flexural strength (MPa)	5.2	-
${Na}_{2}O$	0.18	1.357	28 d Flexural strength (MPa)	7.5	-
$K_{2}O$	0.79	2.170	3 d Compressive strength (MPa)	23.2	-
${SO}_{3}$	2.72	0.620	28 d Compressive strength (MPa)	51.6	-
Ignition loss	1.1	3.1	Activity index (28 d)	-	84%

**Table 2:** Properties of river sands and coarse aggregate.

Properties	Apparent Density (kg/m^3^)	Clay Content (%)	Crush Value (%)	Fineness Modulus
Coarse aggregate	2790	0.60	13.4	-
River sands	2630	1.35	-	2.9

**Table 3:** Mix proportions of concrete mixtures.

Water-Binder Ratio	Mix Proportion/(kg/m^3^)						
	Cement	Fly Ash	Water	Stone	River Sand	SP	AE
0.36	360	65	152	1080	650	5.72	0.76

Note: SP and AE represent polycarboxylate superplasticizer and air-entraining agent.

**Table 4:** Curing conditions of each specimen.

Group	Standard Curing (SC)	Intermediate Curing (IC)	Low-Temperature Curing (LC)
Curing conditions	20 °C—95% RH	5 °C—70% RH	0 °C—50% RH

**Table 5:** Freeze-thaw media for single-sided freeze-thaw test.

Solution	NaCl				${CaCl}_{2}$				KAc				$H_{2}O$
Mass fraction	1%	3%	5%	7%	0.95%	2.85%	4.74%	6.64%	1%	3%	5%	7%	-

**Table 6:** Rate of mass loss, relative dynamic elastic modulus and relative durability index at the end of the test.

Group	Rate of Mass Loss	Relative Dynamic Elastic Modulus	Relative Durability Index	Maximum Number of Freeze-Thaw Cycles
$\mathrm{SC}\text{-}H_{2}O$	5.41	78.81	105.08	400
SC-NaCl	3.51	58.99	29.50	150
$\mathrm{SC}\text{-}{CaCl}_{2}$	3.31	59.34	29.67	150
SC-KAc	4.05	55.57	32.41	175
$\mathrm{IC}\text{-}H_{2}O$	5.19	79.96	93.29	350
IC-NaCl	4.24	53.91	26.96	150
$\mathrm{IC}\text{-}{CaCl}_{2}$	4.08	51.00	25.50	150
IC-KAc	4.78	47.01	27.42	175
$\mathrm{LC}\text{-}H_{2}O$	5.09	71.93	59.94	250
LC-NaCl	4.04	55.83	18.61	100
$\mathrm{LC}\text{-}{CaCl}_{2}$	3.90	51.11	17.04	100
LC-KAc	3.71	59.62	19.87	100

## Data Availability

Data are contained within the article.
